# Associations Between Social Media Engagement and Vaccine Hesitancy

**DOI:** 10.1007/s10900-022-01081-9

**Published:** 2022-03-25

**Authors:** Lola Al-Uqdah, F. Abron Franklin, Chu-Chuan Chiu, Brianna N. Boyd

**Affiliations:** 1 Present Address: Division of COVID Containment, Philadelphia Department of Health, 1101 Market Street, Philadelphia, PA 19107 USA; 2 Epidemiology, Oregon Health & Science University - Portland State University, School of Public Health, Portland, OR 97239 USA; 3 Morehouse School of Medicine, Department of Community Health and Prevention Medicine and Graduate Education in Public Health, Atlanta, GA 30310 USA

**Keywords:** COVID-19 pandemic, Vaccine hesitancy, Social media, Health disparity, Public health

## Abstract

There has been a surge in vaccine hesitancy following the Coronavirus pandemic. This study measured the prevalence of and identified factors associated with vaccine hesitancy and social media use. An online survey was administered (n = 1050) between May and July 2021. Chi-square tests were used to examine bivariate associations with vaccine hesitancy (partially vaccinated and unvaccinated participants). Logistic regression was used to identify associations between social media use and vaccine hesitancy. Chi-square tests showed women (69.7% vs 28.2% men, *p*_*adjusted*_ = .002), African American participants (52.3% vs 17.8% white, *p*_*adjusted*_ < .001), high school diploma (54.4% vs 38.6% college degree, *p*_*adjusted*_ < .001), political unaffiliated (15.8% vs 14.5% republican, *p*_*adjusted*_ < .001), Muslim (10.0% vs 0% Jewish, *p*_*adjusted*_ < .001), and never married/single (53.9% vs 17.0% married, *p*_*adjusted*_ < .001) were more likely to be vaccine hesitant. Controlling for all demographic variables (age, race, gender, and education), more frequent use of social media for reading news was associated with lower vaccine hesitancy (*OR* 0.35, 99% *CI* 0.20, 0.63, *p* < 0.001). However, using social media as a source of vaccine information without any other trusted source (health department, doctor, CDC,) was associated with higher odds of being vaccine hesitant (*OR* 2.00, 99% *CI* 1.15, 3.46, *p* = 0.001). People who use social media without referencing trusted sources may be particularly vulnerable to disinformation or vaccine hesitant persons are more likely exposed to non-trusted social media sites as their only information source.

**Supplementary Information:**

The online version contains supplementary material available at 10.1007/s10900-022-01081-9.

## Introduction

Vaccination has been an essential tool used to fight infectious disease for decades. Diseases such as smallpox and polio have been eradicated after successful worldwide vaccination efforts. In 2019, the COVID-19 virus emerged and has since caused a global pandemic. In response to this crisis, COVID-19 vaccines were developed. Although these vaccines are considered safe, some of the country’s most vulnerable populations (low-income, racial and ethnic minority groups, women and persons without a college degree) responded to these vaccines with distrust [1, 2]. According to the World Health Organization, vaccine hesitancy is indecision, uncertainty, delay and refusal of vaccination despite the availability of vaccination services. Many studies address vaccine hesitancy in general and several factors have been identified in the literature as influencing vaccine hesitancy. These factors include social media, race, gender, age, education, income, family, religion, politics, personal beliefs, trust and experience with the healthcare system [3–7].

Race has consistently been identified as a factor associated with vaccine hesitancy [1, 3, 7]. African Americans in particular distrust the government and the healthcare system [6]. Misinformation about vaccine safety and lack of trust in the healthcare system is also a factor associated with hesitancy even among healthcare workers [7–10]. In fact, a correlation was found between healthcare worker’s belief in vaccine safety and their vaccine recommendations [10]. Other factors such as being female, being younger age, having less education, having lower income, belonging to religious and political groups have all been associated with lower vaccine acceptance rates [1, 2, 7].

Social media use was also identified as a factor influencing vaccine hesitancy [9, 11]. According to Thaker [6], vaccine disinformation is deliberately spread across social media platforms. While most social media sites contain vaccine disinformation and anti-vaccination posts, social media platforms vary in the degree of vaccine disinformation shared with the public [6, 11]. Although vaccine hesitancy existed prior to the COVID-19 pandemic, there has been a recent change in vaccine hesitancy following the Emergency Use Authorizations for the COVID-19 vaccines in the United States [12]. Dube suggests, consuming social media has increased mistrust towards vaccines [12]. Most adults (98%) use social media and it only takes a few minutes of exposure to disinformation on social media to influence vaccine hesitancy. Furthermore, the most vaccine hesitant groups (for COVID-19 vaccines) are frequently the most vulnerable [1, 2, 8]. Therefore, it is imperative for public health officials to understand vaccine hesitancy as it relates to the COVID-19 vaccines and how social media use influences vaccine uptake [6, 11, 13].

This study identified factors associated with vaccine hesitancy, measured activity levels on social media platforms and examined how differences in vaccine information source (family, friends, social media, trusted sources) influence hesitancy. To understand how social media exposure influences vaccine behavior. The objectives of this study were: to measure the prevalence of vaccine hesitancy among vulnerable populations, identify factors associated with vaccine hesitancy among this population, compare activity levels on social media platforms, and determine how vaccine information source (family, friends, social media, trusted sources) influence hesitancy. We hypothesized; (1) More frequent social media use is associated with increased vaccine hesitancy. (2) Vaccine information source will influence vaccine hesitancy (obtaining vaccine information from social media sites will increase vaccine hesitancy).

## Methods

The Philadelphia Department of Public Health Division of COVID Containment developed a questionnaire (The Vaccine Hesitancy and Social Media Use Survey). This questionnaire was a modified version of “The PEW Social Media Use 2021” and “The Social Networking Usage Questionnaire” [13, 14]. This questionnaire was administered from mid-May to early July 2021 and data were collected anonymously via a public self-administered online survey through Qualtrics Panels (a third-party survey panels provider) [1, 15]. The survey instrument captured participants’ demographic information, social media use, sources of vaccine information and vaccine hesitancy. Survey quotas based on race, age and gender were established and participants were selected based on demographics that were representative of Philadelphia’s racial and ethnic demographic distribution (African–American 40%; White 35%; Hispanic 15%; Asian 7%; Other 3%), age (at least 50% age 18 to 44), gender (no more than 50% Females) [16].

A total of 4115 survey responses were attempted online. Survey responses that met our quota and inclusion criteria were included for our data analyses (n = 1050). Inclusion criteria were as follows; (1) persons residing in zip codes located in Philadelphia, (2) ≥ 18 years old, and (3) persons who committed to giving their best answers). The rest of the survey responses (n = 3065) were terminated for not meeting our inclusion criteria (listed above) or for failing the Qualtrics quality check. Qualtrics quality check excluded responses for the following: (1) finishing the survey more than two standard deviations from the mean duration and (2) being identified as a possible bot or duplicate responses). Among the 3065 responses that were excluded, 2402 were terminated by our screening questions (65 were over the quota; 2064 were not located inside a Philadelphia zip code; 17 were under 18 years old; 256 did not commit to provide the best answers). A total of 663 were detected and excluded by Qualtrics quality check system (199 duplicate responses; 49 possible bots; 26 speeders; 389 incomplete).

The survey instrument captured participants’ activity on each social media platform (Twitter, Instagram, Facebook, Snapchat, WhatsApp, LinkedIn, Reddit, Nextdoor, Youtube, Pinterest and TikTok), frequency of use (once a day or more/less than once a day), source of vaccine information (Social Media, Health Care Provider, Centers for Disease Control Website (CDC), City of Philadelphia Website, Local News Major News Network, Friends/Family, Other Website, or Other), Social Media use for Reading News (Always, Sometimes, Rarely/Never), and Vaccine hesitancy (unvaccinated, received 1 dose of a 2 dose vaccine and did not schedule a second dose, unvaccinated and not having any plans to get vaccinated). Trusted sources were Health Care Provider, Centers for Disease Control Website (CDC) and the City of Philadelphia Website. Participants were allowed to choose more than one social media platform and more than one vaccine information source. The complete survey is available in the Supplementary Materials (Table S1).

### Data Analysis

Chi-square tests of independence were used to assess the relationship between all variables (each demographic variable: gender, race, age, education, political affiliation, religion, household status, etc.; social media use variables) and vaccine hesitancy. All analyses were based on two-sided P-values, which statistical significance define by p < 0.01. When the expected frequencies smaller than five, Fisher’s exact test were applied to examine the statical significance [17]. A total of 33 chi-square tests were calculated; for multiple comparisons corrections, we used the Benjamini–Hochberg procedure to decrease the false positive rate/type I error. To present the results of the Benjamini–Hochberg procedure in simple manner, adjusted p values are reported and shown in Tables 1 and 2 [18].

**Table 1:** Demographic, vaccine information source and social media use variables

Demographic variables	Overall N = 1050 (%)^a^	Vaccine information sources variables	Overall N = 1051	
Gender		Social media platform	Yes	No
Female	630 (60.0%)	Twitter	601 (61.0%)	410 (39.0%)
Male	401 (38.2%)	Instagram	830 (79.0%)	220 (21.0%)
Other	19 (1.8%)	Facebook	812 (77.3%)	238 (22.7%)
Age		Snapchat	581 (55.3%)	469(44.7%)
18–24	279 (26.6%)	Youtube	966 (92.0%)	84 (8.0%)
25–34	282 (26.9%)	WhatsApp	452 (43.0%)	598 (57.0%)
35–44	239 (22.8%)	Pinterest	487 (46.4%)	563 (53.6%)
45–54	104 (9.9%)	LinkedIn	456 (38.6%)	645 (61.4%)
55–64	79 (7.5%)	Reddit	283 (27.0%)	767 (73.0%)
66 or older	67 (6.4%)	TikTok	517 (49.2%)	533 (50.8%)
Race and ethnicity		Nextdoor	167 (15.9%)	883 (84.1%)
African-American	392 (37.3%)	Vaccine information source non trusted		
Asian	63 (6.0%)		Yes	No
White	328 (31.2%)	Friends	386 (36.8%)	664 (63.2%)
Hispanic	251 (23.9%)	Family	533 (50.8%)	517(49.2%)
Other/unknown	16 (1.5%)	Social media	352 (33.5%)	698 (66.5%)
Religion		Major News Networks	374 (35.6%)	667 (64.4%)
Agnostic	28 (2.7%)	Other Website	65 (6.2%)	985 (93.8%)
Atheist	30 (2.9%)	Vaccine information source trusted		
Buddhist	17 (1.6%)		Yes	No
Catholic	178 (17%)	Health care provider	514 (49.0%)	536 (51.0%)
Christian	444 (42.3%)	City of Philadelphia	276 (26.3%)	774 (73.7%)
Hindu	6 (0.6%)	CDC website	325 (31.0%)	725 (69.0%)
Jewish	29 (2.8%)	Use social media for reading news		
Muslim	69 (6.6%)	Always	326 (31.0%)	
None	180 (17.1%)	Sometimes	489 (46.6%)	
Other	69 (6.6%)	Rarely/never	235 (22.4%)	
Relationship status		Use social media for sharing new ideas		
Divorced	43 (4.1%)	Always	317(30.2%)	
Married	257 (24.5%)	Sometimes	446(42.5%)	
Never married/living with someone	158 (15.0%)	Rarely/never	287(27.3%)	
Never married/single	438 (41.7%)	Social media frequency of use		
Separated	37 (3.5%)	Several times a day	689 (65.6%)	
Widowed	17 (1.6%)	About once a day	180 (17.1%)	
Education		A few times a week	94 (9.0%)	
High school graduate/trade school/GED	379 (36.1%)	Every few weeks	26 (2.5%)	
College degree	469 (44.7%)	Less often	34 (3.2%)	
Master’s degree or above	202 (19.2%)	I don't know	27 (2.6%)	
Political affiliation				
Democrat	522 (49.7%)			
Republican	230 (21.9%)			
Independent	172 (16.4%)			
Unaffiliated	89 (8.5%)			
Other	37 (3.5%)			

This table displays percentages of demographic variables including race, gender, age, religious affiliation, relationship status and education; vaccine information source variables including social media sites, family, friends, other media, and trusted sources (Healthcare provider, CDC, Health department) and frequency of social media use

^a^The survey was administered between May and July, 2021 (N = 1050)

**Table 2:** Demographic and media variables significantly associated with vaccine hesitancy

Demographic variables	Hesitancy N = 241 (%)^b^	No hesitancy N = 809 (%)^b^	Chi-square test adjusted p value^c^	Media & vaccine information sources variables	Hesitancy N = 241 (%)^b^		No hesitancy N = 809 (%)^b^		Chi-square test adjusted p value^c^
Gender^a^			.002	Social Media Platform	Use	Do not use	Use	Do not use	
Female	168 (69.7%)	462 (57.1%)		Twitter	115 (47.7%)	126 (52.3%)	525 (64.9%)	284 (35.1%)	< .001
Male	68 (28.2%)	333 (41.2%)		Facebook	168 (69.7%)	73 (30.3%)	644 (79.6%)	165 (20.4%)	0.003
Other	5 (2.1%)	14 (1.7%)		Snapchat	118(49.0%)	123(51.0%)	463(57.2%)	346(42.8%)	0.039^d^
Age^a^			< .001	WhatsApp	76 (31.5%)	165 (68.5%)	376 (46.5%)	433 (53.5%)	< .001
18–24	92 (38.2%)	187 (23.1%)		LinkedIn	59 (24.5%)	182 (75.5%)	346 (42.8%)	463 (57.2%)	< .001
25–34	63 (26.1%)	219 (27.1%)		Reddit	40 (16.6%)	201 (83.4%)	243 (30%)	566 (70%)	< .001
35–44	35 (14.5%)	204 (25.0%)		Nextdoor	12 (5%)	229 (95%)	155 (19.2%)	654 (80.8%)	< .001
45–54	25 (10.4%)	79 (9.8%)		Vaccine Information Source Non Trusted	Use	Do not use	Use	Do not use	
55–64	13 (5.4%)	66 (8.2%)							
65 or older	13 (5.4%)	54 (6.7%)		Friends	64 (26.6%)	177 (73.4%)	322 (39.8%)	487 (60.2%)	< .001
Race and ethnicity^a^			< .001	Family	106 (44.0%)	135 (56.0%)	427 (52.8%)	382 (47.2%)	0.029^d^
African-American	126 (52.3%)	266 (32.9%)		Social Media	104 (43.2%)	137 (56.8%)	248 (30.7%)	561 (69.3%)	< .001
Asian	5 (2.1%)	58 (7.2%)		Major News Networks	66 (27.4%)	175 (72.6%)	308 (38.1%)	501 (61.9%)	.005
White	43 (17.8%)	285 (35.2%)		Other Website	25 (10.4%)	216 (89.6%)	40 (4.9%)	769 (95.1%)	0.005
Hispanic	62 (25.7%)	189 (23.4%)		Vaccine Information Source Trusted	Use	Do not use	Use	Do not use	
Other/unknown	5 (2.1%)	11 (1.4%)							
Religion			< .001	Health Care Provider	92 (38.2%)	149 (61.8%)	422 (52.2%)	387 (47.8%)	< .001
Agnostic	3 (1.2%)	25 (3.1%)		City of Philadelphia	29 (12%)	212 (88%)	247 (30.5%)	562 (69.5%)	< .001
Atheist	3 (1.2%)	27 (3.3%)		CDC Website	51 (21.2%)	190 (78.8%)	274 (33.9%)	535 (66.1%)	< .001
Buddhist	0 (0%)	17 (2.1%)		Use Social Media for Reading News					
Catholic	34 (14.1%)	144 (17.8%)			Hesitancy n = 241 (%)		No Hesitancy n = 809 (%)		< .001
Christian	97 (40.2%)	347 (42.9%)		Always	49 (20.3%)		277 (34.2%)		
Hindu	1 (0.4%)	5 (0.6%)		Sometimes	116 (48.1%)		373 (46.1%)		
Jewish	0 (0%)	29 (3.6%)		Rarely/Never	76 (31.5%)		159 (19.7%)		
Muslim	24 (10.0%)	45 (5.6%)		Use Social Media for Sharing New Ideas					
None	57 (23.7%)	123 (15.2%)							< .001
Other	22 (9.1%)	47 (5.8%)		Always	47(19.5%)		270(33.4%)		
Relationship status			< .001	Sometimes	112(46.5%)		334(41.3%)		
Divorced	10 (4.1%)	33 (4.1%)		Rarely/never	82(34.0%)		205(25.3%)		
Married	41 (17.0%)	316 (39.1%)							
Never married/living with someone	42 (17.4%)	116 (14.3%)							
Never married/single	130 (53.9%)	308 (38.1%)							
Separated	13 (5.4%)	24 (3.0%)							
Widowed	5 (2.1%)	12 (1.5%)							
Education			< .001						
High school graduate/trade school/GED	131 (54.4%)	248 (30.6%)							
College degree	93 (38.6%)	376 (46.5%)							
Master’s degree or above	17 (7.0%)	185 (22.9%)							
Political affiliation									
Democrat	100 (41.5%)	422 (52.2%)	< .001						
Republican	35 (14.5%)	195 (24.1%)							
Independent	51 (21.2%)	121 (15.0%)							
Unaffiliated	38 (15.8%)	51 (6.3%)							
Other	17 (7.1%)	20 (2.5%)							

This table displays the demographic and media variables significantly associated with vaccine hesitancy

The survey was administered between May and July, 2021 (N = 1050)

^a^The percentages of Gender, Age, Race and Ethnicity were collected based on quotas similar to the demographic make up of Philadelphia residents as stated in the Methods section

^b^Some percentages do not sum up to 100 because of rounding

^c^Based on Benjamin-Hochberg procedure, with adjusted p value < .01 considered significant

^d^Variables are significant at adjusted p value < .05

Multivariate logistic regression models were built to understand the dynamics between social media use variable and vaccine hesitancy and included the significant variables (gender, race, age, education, social media use) as covariates that were already shown to be related to the outcome (vaccine hesitancy). We considered p < 0.01 to be significant and reported 99% Confidence Interval for odds ratios. Cramer’s V test (a correlation test for categorical variables) was conducted among independent variables to ensure no multicollinearity was observed in the regression models [19]. All results were below 0.28.

## Results

### Demographic Factors Associated with Vaccine Hesitancy

Overall, several demographic and social media factors were significantly associated with vaccine hesitancy. A chi-square test of independence showed gender, age, race, education level, relationship status, political affiliation, and religious affiliation all had a significant association with vaccine hesitancy (*p*_*adjusted*_ < 0.001) (See Table 2). A surprising finding was that religious affiliation can have both a negative and a positive correlation to vaccine hesitancy. Participants who identified as Jewish are significantly less likely to be hesitant (*p*_*adjusted*_ < 0.001). Whereas participants who identified as Muslim other religion or no religious affiliation are significantly more likely to be hesitant (*p*_*adjusted*_ < 0.001). Receiving vaccine information from friends (*p*_*adjusted*_ < 0.001) and family were found to be significant (*p*_*adjusted*_ = *0.029)* (See Table 2).

### Vaccine Hesitancy and Social Media Use

**Fig. 1:**
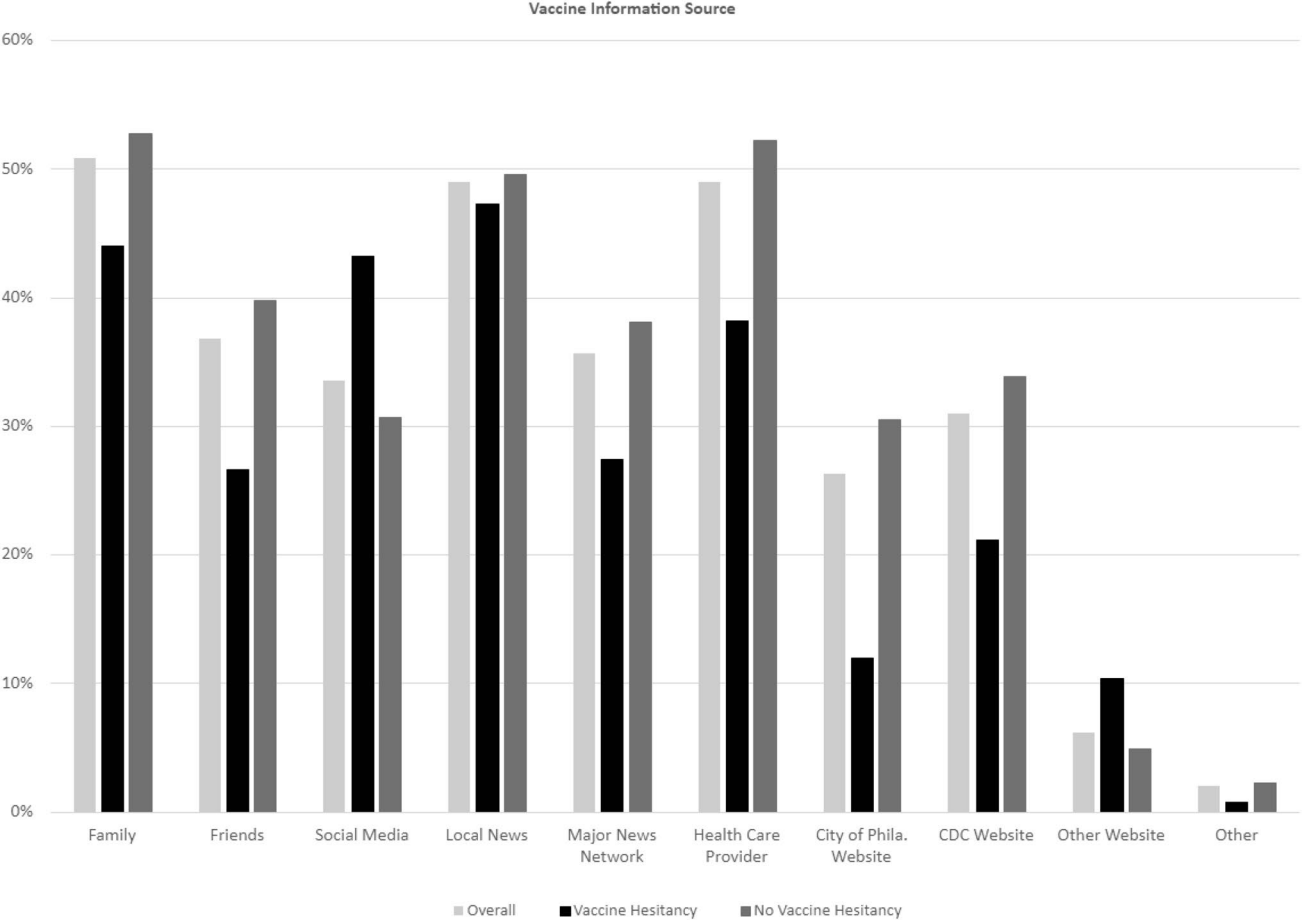
Vaccine information. *Source* This graph shows the relationship between vaccine information source and vaccine hesitancy. Obtaining vaccine information from non-trusted social media sites is predictive of vaccine hesitancy

**Fig. 2:**
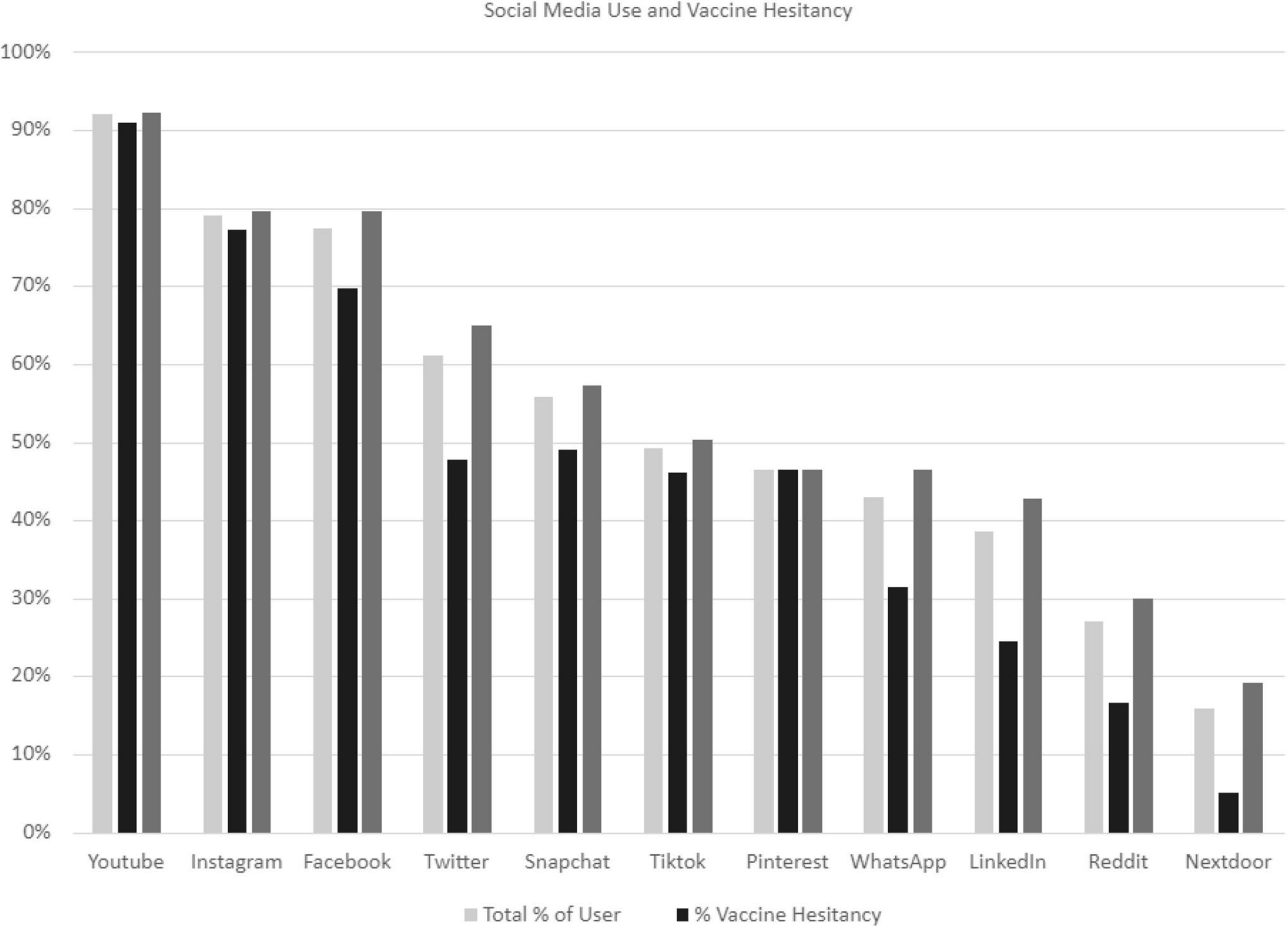
Social media use and vaccine hesitancy. This graph shows the relationship between social media use and vaccine hesitancy

Among the Never Vaxer (NV) hesitancy group “No, I do not want to be vaccinated now or at any-time in the future”, Instagram is the second most widely used platform among the age group 18–34 in both the (NV) and the (SV) “No, I do not want to be vaccinated at this time” group. Among the age group 35 and 54, Facebook was the second most popular platform after YouTube in both the (NV) and in the (SV) groups. Among age 55 or older, YouTube and Facebook remain the most popular platforms in both the NV and SV groups (Figs. 1, 2).

Our results did not support hypothesis number 1. More frequent social media use is associated with increased vaccine hesitancy. Controlling for all demographic variables, more frequent use of social media for reading news was associated with lower odds of being vaccine hesitancy (*OR* 0.35, 99% *CI* 0.20, 0.63, *p* < 0.001). Therefore, the data are not shown. The data does however support hypothesis number 2. Vaccine information source does influence vaccine hesitancy (obtaining vaccine information from non-trusted social media sites will increase vaccine hesitancy), Using social media as a source of vaccine information without any other trusted source (health department, doctor, CDC,) was associated with higher odds of being vaccine hesitant (*OR* 2.00, 99% *CI* 1.15, 3.46, *p* = 0.001) (See Table 3).

**Table 3:** Multivariable logistic regression results

Independent variable	Adjusted odds ratio (99% Confidence Interval) [*P* Value]	
	Model 1: Use Social Media for Vaccine Information	Model 2: Frequency of Using Social Media for Reading News
Age		
18–24	1 [Reference]	1 [Reference]
25–34	0.79 (0.46, 1.36) [0.270]	0.87 (0.50, 1.49) [0.495]
35–44	0.61 (0.32, 1.14) [0.041]	0.62 (0.33, 1.16) [0.048]
45–54	0.91 (0.44, 1.89) [0.748]	0.81 (0.39, 1.68) [0.45]
55–64	0.54 (0.22, 1.33) [0.078]	0.44 (0.18, 1.08) [0.018]
65+	0.75 (0.30, 1.91) [0.433]	0.53 (0.21, 1.36) [0.085]
Race		
White	1 [Reference]	1 [Reference]
African American	1.99 (1.12, 3.52) [0.002]	2.15 (1.21, 3.82) [0.001]
Asian	0.46 (0.12, 1.74) [0.136]	0.47 (0.12, 1.79) [0.147]
Hispanic	1.41 (0.75, 2.66) [0.162]	1.56 (0.82, 2.95) [0.073]
Other/unknown	2.41 (0.51, 11.41) [0.144]	2.77 (0.58, 13.16) [0.092]
Gender		
Female	1 [Reference]	1 [Reference]
Male	0.87 (0.55, 1.40) [0.461]	0.87 (0.54 1.39) [0.447]
Other	0.64 (0.15, 2.66) [0.415]	0.6 (0.14, 2.61) [0.375]
Highest education		
College degree	1 [Reference]	1 [Reference]
High school degree	1.72 (1.11, 2.65) [0.001]	1.7 (1.10, 2.63) [0.002]
Master’s or above degree	0.43 (0.20, 0.90) [0.003]	0.42 (0.20, 0.89) [0.003]
Use social media for vaccine information^a^		
Not use social media	1 [Reference]	
Use social media with trusted sources	1.16 (0.69, 1.93) [0.458]	
Use social media alone or with other non-trusted sources	2 (1.15, 3.46) [0.001]	
Using social media for reading news		
Rarely/never		1 [Reference]
Always		0.35 (0.20, 0.63) [<0.001]
Sometimes		0.65 (0.40, 1.07) [0.027]

Model 1 examined the association between vaccine hesitancy and social media use for vaccine information while controlling the selected variables constanta; Model 2 examined the association between vaccine hesitancy and the frequency of using social media for reading news while controlling the selected variables constanta (N = 1050)

Predictor Variables used in the regression models include Age, Race, Gender, and Highest Education

^a^Variables were regrouped into 3 categories (do not use social media, use social media with trusted sources, use social media alone or with other non-trusted sources) to better understand how social media use influences vaccine hesitancy

## Discussion

The purpose of this study was to measure the prevalence of vaccine hesitancy among vulnerable populations, identify factors associated with vaccine hesitancy among this population, compare activity levels on social media platforms, and determine how vaccine information source (family, friends, social media, trusted sources) influence s hesitancy. We hypothesized that more frequent social media use is associated with vaccine hesitancy. Our findings did not support this hypothesis. This may be because we had insufficient data to detect an association. Our findings did support hypothesis number 2. Our study found that vaccine information source (non-trusted social media sites) influences vaccine hesitancy. We found that frequenters of non- trusted sites are more likely to be hesitant and visitors of trusted sites such as CDC, PDPH and healthcare providers, are less likely to be hesitant. Our results indicate that social media can be a good source of vaccine information when the information is delivered by someone considered to be a trusted source in the community [8]. Health care providers, health departments and the CDC are considered trusted sources [11]. Trusted social media sites provide accurate fact-based information. Whereas many non-trusted social media sites do not provide factual-information or scientific evidence supporting their claims [11]. Our findings suggest that people who use social media alone without referencing trusted sources may be particularly vulnerable to disinformation or that vaccine hesitant persons are more likely to have been exposed to non-trusted social media sites as their only source of vaccine information. hesitant are especially drawn to social media as their only information source. Furthermore, social media users may engage in confirmation bias by seeking out vaccination information that supports their pre-conceived beliefs. Additional studies reveal that many social media sites contain vaccine disinformation and anti-vaccination posts [11]. Pro and Anti-vaccination social media users are often siloed on social media and anti-vaccination post on social media is often re-posted or re-tweeted more frequently that neutral posts and tweets [11, 20, 21]. Although, the effects of viewing non-factual vaccine information or even vaccine disinformation information on less trust-worthy social media sites appears to be mitigated in better-educated, older, less racially diverse social media users who also view vaccine information on trusted social media sites.

While viewing vaccine information on trusted social media sites may dilute the effects of vaccine disinformation for some, younger adults, African American persons, and less educated cohorts appear to be more susceptible to vaccine disinformation [11] found that some social media users, such as those who are less educated are highly susceptible to social media campaigns. Women, African American persons, persons ages (18–44), less educated persons, those who identify as Muslim, no religious affiliation or other, those who are single never married, single living with someone, or separated and those who are politically unaffiliated are more likely to be hesitant. This finding has important implications. There is a correlation between age and social media use. Over 80% of adults between the ages of 19 and 34 use social media and adults ages 18 to 39 display greater social media use when compared to other age groups [13]. Furthermore, the Household Pulse Survey Covid-19 Vaccination Tracker found that persons aged 25 to 39 display greater vaccine hesitancy for COVID-19 vaccine uptake than any other age group. African American persons have a long history of being victims of systemic racism and of being ignored or exploited by the government and the medical community. This lived experience fuels African American persons’ distrust in the vaccine and is often cited as the reason for hesitancy [8, 22]. Reasons for hesitancy in less educated cohorts may have more to do with health literacy. Some persons may have difficulty understanding the science or navigating the ever- changing COVID-19 guidelines and may be more responsive to less factual emotional appeals on social media [11]. Thus, the need for pro-active simple easy to read social media content about vaccine safety, efficacy, and side effects [11].

While talking with family and friends about vaccines is associated with decreased vaccine hesitancy, being single never married, single living with someone, or separated is associated with increased vaccine hesitancy. In other words, our findings support the notion that social pressure is effective. Family and friends (social pressure) can convince their loved ones to get vaccinated [8]. It may also be the cases that persons who live alone or who are unmarried may experience less social pressure in the home which may explain why they are more likely to be hesitant. Our study also found that politically unaffiliated persons and Independents are more likely to be hesitant. This finding is not supported in the literature. A recent study by Kaiser Family Foundation found that Republicans were more likely to be vaccine hesitant [23]. Persons who identify as Jewish or Buddhist are least likely to be hesitant and persons who identify as Muslim or other religion are more likely to be hesitant. Faith Based Organizations (FBOs) can be effective facilitators in reducing vaccine hesitancy and increasing vaccine uptake because clergy members have significant influence on congregation members’ health behaviors and FBOs provide the type of social support that encourages and significantly improves healthy behaviors [24]. Additional studies demonstrate that most people would like to hear information about vaccine efficacy from scientists and not from lay men or community leaders [11]. Therefore, addressing the concerns that specific communities of faith have about the COVID-19 vaccines is a critical step in the fight to reduce vaccine hesitancy [25–27].

As public health officials if we want to reduce vaccine hesitancy, we must address hesitancy where it occurs. Identifying the specific demographic make-up of vaccine hesitant populations can inform future targeted public health campaigns. Understanding that racial groups are not monolithic and that there is no one size fits all is key. People within the same racial group have different perspectives and motives for hesitancy across other demographics. There is a need to specifically target the reason for hesitancy within each of these subgroups. Lower vaccination rates in African Americans and in low-income communities can increase health inequities and lead to further disparities. Therefore, efforts to combat vaccine hesitancy must be tailored to fit each of these subgroups. Reducing vaccine hesitancy is a necessary step toward reducing health disparities resulting from the COVID-19 virus and achieving health equity [1, 2]. If we can increase vaccine uptake in vulnerable communities, we can move one step closer to achieving herd immunity.

## Implications for Policy and Practice

- Our research shows several factors are significantly associated with vaccine hesitancy. Age (18 to 34), Women, African American persons, persons without a bachelor’s degree and individuals who use social media for vaccine information are more likely to be hesitant.
- Over 93% of the participants surveyed use social media, over 98% of vaccine hesitant participants use social media, and persons aged (18 to 39) use social media more than any other age group.
- Based on our study results it imperative that public health entities reduce vaccine hesitancy by mounting targeted campaigns on social media platforms in populations where hesitancy frequently occurs.
- Social media platforms vary in the degree of vaccine disinformation shared with the public, therefore, there is a need for proactive communication strategies to respond to misinformation on social media by specifically tailoring communications to each social media platform and its users [11].
- There are race-based inequities in the healthcare system [8] and a general mistrust of the government. Thus, sincere long-term efforts should be made to establish trust between the most vulnerable communities, the government and health care system by establishing permanent relationships with community-based organizations that persist after the pandemic.

## Study Limitations

The data collection of the study was conducted by a Qualtrics panel survey. That implies our survey may not adequately sample persons with low technological abilities. In addition, our study was cross-sectional and collected at one specific time. Factors influencing vaccine hesitancy may change over time.

## Recommendations for Future Studies

We offer the following recommendations to reduce vaccine hesitancy and to increase vaccine equity.

1. We plan a future study with a larger sample size to better understand how frequency of social media engagement influences vaccine hesitancy.
2. We also plan a future study examining factors influencing booter hesitancy.
3. Additional studies are warranted to determine the effect of full FDA approval of the COVID-19 vaccines and the implementation of mandates on vaccine changes the associations observed in our study.

## Supplementary Information

Below is the link to the electronic supplementary material.

Supplementary file1 (PDF 177 kb)

## Data Availability

The data that support the findings of this study are available from [The Philadelphia Department of Public Health] but restrictions apply to the availability of these data and are not publicly available. Data are however available from the authors upon reasonable request and with permission of [The Philadelphia Department of Public Health].
