# Supplementary material for: Associations Between Social Media Engagement and Vaccine Hesitancy
Source: J Community Health. 2022 Mar 25;47(4):577–87. doi: 10.1007/s10900-022-01081-9 (PMC8947854; doi:10.1007/s10900-022-01081-9)
Supplement: Supplementary file 1 — Supplementary file1 (PDF 177 kb) [file 10900_2022_1081_MOESM1_ESM.pdf]

Social Media and Vaccine Hesitancy

Start of Block: Qualtrics Surveys Question Demo

Introduction
To help control the spread of COVID-19 and protect the health of Philadelphians, the Philadelphia Department of Public Health (PDPH) is contacting individuals to better understand the public's vaccine experiences and related factors. Your answers are completely voluntary and confidential.

We care about the quality of our survey data and hope to receive the most accurate measures of your opinions, so it is important to us that you thoughtfully provide your best answer to each question in the survey.

Q1 **Do you commit to providing your thoughtful and honest answers to the questions in this survey?**

○ I will provide my best answers. (1)

○ I will not provide my best answers. (2)

○ I can't promise either way. (3)

Skip To: End of Block If Q1 = 3

Skip To: End of Block If Q1 = 2

Q2 **What 5-digit zip code do you live in Philadelphia?**

____

Skip To: End of Block If Condition: What 5-digit zip code do yo... Does Not Contain 191. Skip To: End of Block.

Q3 **What gender do you identify as?**

- ◯ Male (1)
- ◯ Female (2)
- ◯ Transgender Male (3)
- ◯ Transgender Female (4)
- ◯ Non-binary / third gender (5)
- ◯ Self describe gender (6) ____

Q4 **What is your age?**

- ◯ Under 18 years old (1)
- ◯ 18 - 24 (2)
- ◯ 25 - 34 (3)
- ◯ 35 - 44 (4)
- ◯ 45 - 54 (5)
- ◯ 55 - 64 (6)
- ◯ 65 or older (7)

Skip To: End of Block If Q4 = 1

Q5 **Please specify your race.**

- ○ Caucasian (1)
- ○ African-American (2)
- ○ Latino or Hispanic (3)
- ○ Asian (4)
- ○ Native American (5)
- ○ Native Hawaiian or Pacific Islander (6)
- ○ Two or More (7)
- ○ Other/Unknown (8)

Q6 **Please specify your ethnicity.**

- ○ Hispanic (1)
- ○ Non- Hispanic (2)

Q7 **What is your primary language?**

- English (1)
- Spanish (2)
- Portuguese (3)
- French (4)
- Mandarin (5)
- Arabic (6)
- Russian (7)
- Other (8) ____

Q8 **What is your present religion, if any?**

- Agnostic (1)
- Atheist (2)
- Buddhist (3)
- Catholic (4)
- Christian (5)
- Hindu (6)
- Jewish (7)
- Muslim (8)
- None (9)
- Other (10) ____

Q9 **How many people live in your household (including yourself)?**

[Count everyone living and sleeping in your home most of the time, including young children, roommates, and friends and family members who are living with you, even temporarily.]

- ○ 1 - I live alone (1)
- ○ 2 (2)
- ○ 3 (3)
- ○ 4 (4)
- ○ 5 (5)
- ○ 6 or more (6)

Q10 **Please select the option that describes your current relationship status.**

- ○ Married (1)
- ○ Divorced (2)
- ○ Separated (3)
- ○ Widowed (4)
- ○ Never married/ living with someone (5)
- ○ Never married/ single (6)

Q11 **What is the highest degree or level of education you have completed?**

- ○ Some High School (1)
- ○ High School Graduate / Trade School / GED (2)
- ○ Some College (3)
- ○ Bachelor's Degree (4)
- ○ Master's Degree (5)
- ○ Medical , Law, or Doctoral Degree (6)

Page Break

Q12 **How would you describe your political affiliation?**

- ○ Democrat (1)
- ○ Republican (2)
- ○ Independent (3)
- ○ Unaffiliated (4)
- ○ Other (5) ____

Q13 **Please tell me if you ever use any of the following online platform/app.**
Do you ever use ___? (Once you click "Yes" or "No" on your screen, the name of the platform/app in the box will automatically change to the next until you reach the last one named "Nextdoor.")

	Yes (1)	No (2)
Twitter (1)	○	○
Instagram (2)	○	○
Facebook (3)	○	○
Snapchat (4)	○	○
YouTube (5)	○	○
WhatsApp (6)	○	○
Pinterest (7)	○	○
LinkedIn (8)	○	○
Reddit (9)	○	○
TikTok (10)	○	○
Nextdoor (11)	○	○

Q14 **Thinking about the social media sites or mobile apps you use, how often do you visit or use?**

- Several times a day (1)
- About once a day (2)
- A few times a week (3)
- Every few weeks (4)
- Less often (5)
- I don't know (6)

Q15 **Thinking about the social media sites or mobile apps you use, how often do you use social networking sites for reading news?**

- Always (1)
- Sometimes (2)
- Rarely (3)
- Never (4)

Q16 **Thinking about the social media sites or mobile apps you use, how often do you use social networking sites for sharing new ideas?**

- Always (1)
- Sometimes (2)
- Rarely (3)
- Never (4)

Page Break

Q17 **Have you been vaccinated for COVID-19?**

- ○ Yes, I am fully vaccinated (both doses of a two-dose vaccine or one dose of a single-dose vaccine) (1)
- ○ Yes, I am partially vaccinated. I have received one dose of a two-dose vaccine (2)
- ○ No, but I am scheduled for an appointment (3)
- ○ No, but I would like to schedule an appointment (4)
- ○ No, I do not want to be vaccinated at this time (5)
- ○ No, I do not want to be vaccinated now or at anytime in the future (6)

Display This Question:

If Q17 = 2

Q17-1 **Do you plan on getting the second dose of the vaccine?**

- ○ Yes (1)
- ○ No. Please explain why. (2) ____
- ○ Maybe (3)

Q18 **Where do you get information about vaccines? Select all that apply.**

- ☐ Family (1)
- ☐ Friends (2)
- ☐ Social media (3)
- ☐ Local news (4)
- ☐ Major news networks (5)
- ☐ Health care provider (Doctor, Nurse, Pharmacist) (6)
- ☐ The City of Philadelphia website (7)
- ☐ The Centers for Disease Control (CDC) website (8)
- ☐ Other websites (9)
- ☐ Other, please specify (10) ____

Q19 **What barriers to vaccination, if any, have you experienced? Select all that apply.**

- ▢ Unable to find a vaccination location (1)
- ▢ Cannot go to appointments during the day (work, school, childcare, and other commitments) (2)
- ▢ Unable to go without assistance (3)
- ▢ No transportation to vaccine clinics (4)
- ▢ Pressure from those around you not to be vaccinated (5)
- ▢ Concerns about vaccine safety and/or side effects (6)
- ▢ I have not experienced any barriers to vaccination (7)
- ▢ Other, please specify (8) ____

Display This Question:

If Q17 = 3

Or Q17 = 4

Or Q17 = 5

Or Q17 = 6

Or Q17-1 = 2

Or Q17-1 = 3

Q20 **Please indicate your level of agreement with the following choices.**
"I am more likely to get vaccinated if _______."

(You can simply click "Strongly agree", "Agree", "Somewhat agree", "Neither agree nor disagree", "Somewhat disagree", "Disagree", "Strongly disagree" and then the statement will automatically move to the next. Or, you can click ">" to move to the next statement.)

	Strongly agree (1)	Agree (2)	Somewhat agree (3)	Neither agree nor disagree (4)	Somewhat disagree (5)	Disagree (6)	Strongly disagree (7)
Vaccinations were offered where you work (1)	o	o	o	o	o	o	o
Your employer gave you the day off to get vaccinated. (2)	o	o	o	o	o	o	o
A vaccination site was located within walking distance of your home (3)	o	o	o	o	o	o	o
A vaccination site was open 24 hours (4)	o	o	o	o	o	o	o
Transportation to a vaccination site was provided (5)	o	o	o	o	o	o	o
You are given a gift card to get vaccinated (6)	o	o	o	o	o	o	o
You are given money to get vaccinated (7)	o	o	o	o	o	o	o
I do not plan to get	o	o	o	o	o	o	o

vaccinated now or at anytime in the future (8)

End of Block: Qualtrics Surveys Question Demo
